# Customization scenarios for de-identification of clinical notes

**DOI:** 10.1186/s12911-020-1026-2

**Published:** 2020-01-30

**Authors:** Tzvika Hartman, Michael D. Howell, Jeff Dean, Shlomo Hoory, Ronit Slyper, Itay Laish, Oren Gilon, Danny Vainstein, Greg Corrado, Katherine Chou, Ming Jack Po, Jutta Williams, Scott Ellis, Gavin Bee, Avinatan Hassidim, Rony Amira, Genady Beryozkin, Idan Szpektor, Yossi Matias

**Affiliations:** 1 Google Research, Google LLC, 1600 Amphitheatre Parkway, Mountain View, CA USA; 2 Palo Alto, CA USA

**Keywords:** De-identification, Electronic health records, Free text, Clinical notes, Natural language processing, Recurrent neural networks

## Abstract

**Background:**

Automated machine-learning systems are able to de-identify electronic medical records, including free-text clinical notes. Use of such systems would greatly boost the amount of data available to researchers, yet their deployment has been limited due to uncertainty about their performance when applied to new datasets.

**Objective:**

We present practical options for clinical note de-identification, assessing performance of machine learning systems ranging from off-the-shelf to fully customized.

**Methods:**

We implement a state-of-the-art machine learning de-identification system, training and testing on pairs of datasets that match the deployment scenarios. We use clinical notes from two i2b2 competition corpora, the Physionet Gold Standard corpus, and parts of the MIMIC-III dataset.

**Results:**

Fully customized systems remove 97–99% of personally identifying information. Performance of off-the-shelf systems varies by dataset, with performance mostly above 90%. Providing a small labeled dataset or large unlabeled dataset allows for fine-tuning that improves performance over off-the-shelf systems.

**Conclusion:**

Health organizations should be aware of the levels of customization available when selecting a de-identification deployment solution, in order to choose the one that best matches their resources and target performance level.

## Introduction

Over the past several years, health service researchers have significantly expanded their use of free text in medical research [1]. For example, between 2007 and 2018, the number of PubMed records with “free text” or “unstructured text” more than tripled [2]. Advances in natural language processing and machine learning, and access to de-identified clinical datasets, have contributed to this increase [3].

De-identified clinical datasets are created by labeling all words and phrases that could identify an individual, and replacing them with surrogate data or context-specific labels. For example, “John London complains of chest pain that started on January 1st 2012” becomes “[PersonNameTag] complains of chest pain that started on [DateTag]”. The de-identification process needs to have high recall (sensitivity) since publicly releasing text containing protected health information (PHI) represents a legal and ethical liability. On the other hand, it also needs to have reasonable precision, because unnecessarily removing non-identifying text limits the data’s usefulness to researchers [4]. Notice also that the de-identification system needs to be context-aware: London, usually a location, is accurately labeled a name based on the sentence structure.

Automatic de-identification systems have not been widely adopted on a commercial level, despite the fact that their performance already surpasses that of human annotators: fully customized de-identification systems achieve precision and recall of 97% or higher [5, 6], while the average human recall and precision are 81 and 98% respectively [7].

A blocking factor is that, like many other tools based on machine learning, a de-identification system cannot *guarantee* performance on all medical text it will ever encounter. Structured data such as forms are easy to de-identify, e.g., by removing “Name” and “Date” fields; however, free-text clinical notes vary widely with note purpose and institutional conventions, and include PHI in ways that are challenging to identify and redact.

This challenge may necessitate customizing the de-identification system in order to teach it about the formatting and jargon used in a particular organization. Consider a note with the line “John referred to Alzheimer’s clinic ...”. An off-the-shelf system knows “Alzheimer’s” as a medical condition and de-identifies to “[PersonNameTag] referred to Alzheimer’s clinic...”; a system customized on a target organization’s labeled data where Dr. Alzheimer and his clinic appear frequently, would correctly give “[PersonNameTag] referred to [PersonNameTag]’s clinic...”.

As reviews of de-identification strategies in healthcare concluded, “True anonymization is challenging, and further work is needed in the areas of de-identification of datasets” [8] and “de-identification is not yet a solved problem.” [9]

Therefore, in such a privacy-sensitive field, healthcare organizations need to employ de-identification systems in a controlled manner, with performance assurances specific to each deployment. In order to help organizations make an informed decision, we use publicly available clinical note datasets to assess the performance of automated de-identification systems in several deployment scenarios.

Our first scenario is a fully customized system: a healthcare organization employs human annotators to label a sufficiently large number of PHI examples for training a new machine learning model to perform automated de-identification. We assess performance in this scenario using 3 medical datasets, training the model on part of a dataset and evaluating on the remainder of the dataset.

Our second scenario is off-the-shelf use: the organization provides no labeled data, instead using a pre-trained model as-is. We replicate this scenario by training custom models on 3 datasets and testing each model on all other datasets.

Our third scenario is partial system customization with labeled data: the organization has the resources to provide *some* labeled data. Since labeled data is an expensive resource, requiring the work of human annotators, we study how many labeled examples are needed to improve performance over the off-the-shelf scenario, and how many are required to obtain results equivalent to the fully customized scenario.

Our last scenario is partial system customization with unlabeled data: the organization can avoid the legal and privacy concerns involved with annotating data, and instead improve performance using a large set of its *unlabeled* data. With this data we create a custom token embedding (data representation) for the machine learning system.

## Related work

The first automated approaches for medical free-text de-identification were proposed in the late 1990s and were mainly rule-based [10, 11]. Subsequent work applied machine-learning algorithms and statistical methods such as decision trees [12] and support vector machines [13–15]. These methods required substantial feature-engineering efforts. In the last few years, techniques have shifted towards artificial neural networks and in particular deep neural networks; Yogarajan et al. review current trends [16]. Dernoncourt et al. [5] were the first to use artificial neural networks directly for de-identification of medical texts, showing improved performance. Recently, artificial neural networks were used in several studies, often in combination with rule-based heuristics [6, 17, 18]. Although in practice heuristics are recommended [19], in our work we choose not to use them in order to isolate the contribution of the machine learning model.

Our partial customization scenario with labeled examples is an example of semi-supervised transfer learning/domain adaptation; we build on the work of Lee JY et al. in neural networks [20]. Lee H-J et al. compare 3 transfer learning techniques for de-identification [21]. Kim et al. study questions similar to ours but for concept extraction from notes, also concluding that transfer learning improves performance of a general model [22]. Our partial customization scenario using unlabeled examples falls under unsupervised domain adaptation, techniques for which include domain-specific embeddings [23] and propensity score weighting [24]. Our off-the-shelf scenario serves as a baseline for both adaptation scenarios.

## Methods

### Data sources

The US HIPAA de-identification standard specifies the use of either “Expert Determination” or the “Safe Harbor” method to de-identify data [25]. In the Safe Harbor method, 18 types of patient PHI are removed (Name, Address, Day & Month, Age over 89, Telephone, etc). We use publicly available datasets of de-identified clinical records meeting the Safe Harbor criteria. These datasets were de-identified by replacing PHI with plausible but realistic surrogate information; we evaluate our systems on this surrogate PHI. Throughout the paper the term PHI is used to mean such surrogate PHI.

From the i2b2 National Center for Biomedical Computing for the NLP Shared Tasks Challenges, we use the i2b2-2006 [26] and i2b2-2014 [9, 27] datasets. The i2b2-2006 de-identification guidelines conform to the Safe Harbor standard and further add hospital and doctor name to the list of removed identifiers; the i2b2-2014 guidelines are even more risk averse, removing also e.g. all years [27]. Before release, these datasets were hand-labeled and surrogated.

We also use the PhysioNet gold standard corpus of de-identified medical text [28], containing surrogate information; annotators generated the labeling in-house following the i2b2-2014 guidelines.

Finally, we use the Medical Information Mart for Intensive Care III (MIMIC-III) dataset [29]. This dataset was de-identified before release using the PhysioToolkit deid software package, which expands on Safe Harbor to include ethnicities, clinical provider numbers, and other indirect identifiers [7]. The PHI identification process was based on regular expressions, and has a substantial number of false positives [5]. Our annotators replaced detectable false positives with plausible text. The remaining placeholders were replaced with fictitious values from “real world” distributions consistent with the PHI type specified in the placeholder. We generated three subsets from the MIMIC-III corpus: mimic3-radiology, mimic3-echo, and mimic3-discharge, each containing 1000 notes of the prescribed type.

The i2b2-2006, i2b2-2014, and Mimic-III de-identification guidelines vary regarding which entities are considered PHI. When dataset pairs in an experiment were annotated with the same guideline, we report results on all PHI types in the guideline; in cross analyses, we use the Name PHI, which is labeled consistently across all guidelines. The train/test splits were made by patient. The i2b2 datasets were released with a supplied partition, and the remaining datasets were split randomly. Descriptive statistics are given in Table 1.

**Table 1:** Descriptive statistics for datasets. Mimic3-echo does not contain enough PHI on which to train a model, and is thus used for testing only. We select Name, Date, and Location to show the variety in frequency of PHI types within the datasets

Dataset	Note source	# of patients	# of notes	Train/Test partition by note	Total tokens	Total PHIs	% NAME	% DATE	% LOCATION
i2b2-2014	diabetic longitudinal records	296	1304	61% / 39%	758 k	28.8 k	24.2%	43.3%	15.2%
i2b2-2006	discharge notes	889	889	75% / 25%	487 k	19.5 k	24.0%	36.4%	13.7%
physionet	nursing notes	163	2434	59% / 41%	345 k	1.9 k	32.5%	29.7%	25.9%
mimic3-radiology	radiology notes	1000	1000	50% / 50%	205 k	4.1 k	10.2%	44.8%	1.8%
mimic3-echo	echocardiogram notes	1000	1000	Test only	276 k	2.5 k	9.7%	88.7%	1.1%
mimic3-discharge	discharge notes	1000	1000	81% / 19%	128 k	40.8 k	21.2%	61.1%	9.9%

### Text De-identification system architecture

Our machine learning model implements the state-of-the-art in de-identification of medical notes [5, 6] and named entity sequence tagging [30]. In our analyses, however, any sufficiently powerful model could be substituted. The unbiased recall/precision/F1 of our system on i2b2-2014 (97.1/98.3/97.7) is on par with Dernoncourt et al. [5] (97.4/98.3/97.8), Liu et al. [6] (97.5/98.7/98.1), and the open-source NeuroNER [31] (F1 of 97.7).

**Fig. 1:**
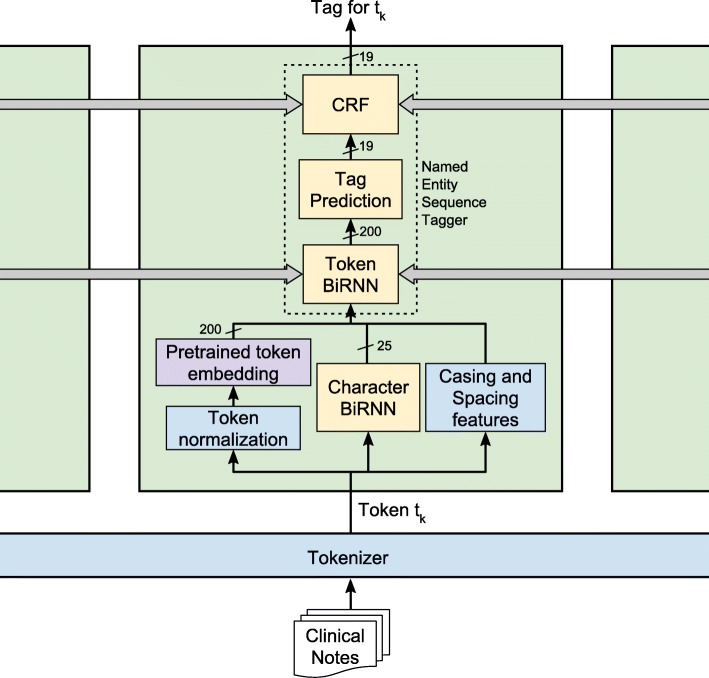
Our de-identification system architecture. Clinical notes are broken into tokens, which are run through the network to be tagged as Not-PHI or Name, Date, etc.

Figure 1 depicts our high level system design, with the green block repeated for each token in the sequence. Yellow blocks require training by labeled examples, purple blocks require training on large numbers of unlabeled examples, and light blue blocks are hardcoded rules. The architecture consists of the following blocks:

1. A Tokenizer, breaking down the input text into tokens, e.g. “Patient prescribed 50mg...” is split into (*Patient, prescribed, 50, mg, ...*).
2. vToken normalization, converting characters to lowercase, and digits to zero.
3. A Pretrained token embedding, mapping each token into a 200-dimensional vector space. We use either GloVe [32] or a custom mapping.
4. A Character BiRNN, generating a corpus-specific, character-based token embedding into a 25-dimensional vector space. This mapping augments the token embedding by learning corpora-specific token and sub-token patterns. This augmentation helps to tackle out-of-vocabulary words, abbreviations, and common prefix/suffix information.
5. A casing feature, giving information about the token’s casing (upper, lower, mixed capitalization), and the number of spaces and line breaks before the token.
6. A Named Entity Sequence Tagger, responsible for converting a token sequence to a tag sequence while taking into account context information. For example, in the sentence “Mr. Jack London was examined”, “London” should be tagged as a person’s name. The Tagger consists of the following:

- A Token BiRNN, adding context information to the extracted token information.
- A Tag prediction layer, projecting the 200 dimensional BiRNN into a probability distribution over the PHI tags (name, age, location, other, etc), including the “not PHI” tag.
- A Conditional Random Field, imposing an additional structured prediction layer to make sure that PHI labels make sense as a sequence.

Model training was carried out using cross entropy loss over the given set of labeled examples as the loss function, and applying Adagrad [33] as the stochastic gradient update function with a batch size of 20. We also applied dropout in layers 1 and 2.

Evaluation results are given for recall (percent of detected PHI out of total PHI), precision (percent of detected PHI which was indeed PHI), and F1 score (the harmonic mean of recall and precision). We can tune the balance between recall and precision by setting the activation bias for “not PHI” prior to the Conditional Random Field block. In an off-the-shelf system, this tuning would be accomplished using heuristics or manual monitoring.

Because recall is of highest importance for patient privacy, we compare system recall using R@P as described in, e.g., Eban et al. [34, 35] The metric takes the highest recall for precision > = p (we select *p* = 85%), while not adjusting if precision is less than this cutoff. As shown in Table 1, the data is heavily imbalanced towards non-PHI tokens; therefore, even a low-precision de-identification system retains the vast majority of non-PHI tokens.

### Experimental setup for deployment scenarios

We perform four experiments using the text de-identification system described in the previous section.

#### A fully customized system

We follow the standard machine learning setup of training and testing on the same dataset “A”.

#### An off-the-shelf system

We follow the standard machine learning setup of training on a dataset “A” and testing on a different dataset “B”.

#### Partial customization using a small number of labeled examples

We train the system on “A” augmented with “n” labeled PHI instances from “B”, and test on “B”. We consider three ways to make use of the additional “n” samples from “B”:

1. Train from scratch: A new model is trained using these “n” datapoints. We call this model “only B”.
2. Further tune an existing model: A model pre-trained on “A” receives further training on “B”. We call this model “A then B”.
3. Jointly train a new model: A model is trained from scratch using an even mixture of “A” and “B”. We call this model “A mix B”.

#### Partial customization using a large number of unlabeled examples

We train the system on “A” only, using a custom token embedding that is generated using unlabeled dataset “B”. A token embedding maps a discrete word to a floating-point vector; vectors corresponding to similar words cluster together, thus providing information about the language to the machine learning system. Token embeddings are built using large unlabeled text corpora; in some settings, using domain-specific corpora improves system performance [36, 37]. We replace the generic GloVe [32] token embedding (2.2 M unique tokens) used in the first three scenarios with custom embeddings built using the word2vec algorithm [38, 39], using tokens (words) that appear at least 10 times. We build embed-mimic (2 M notes, 101 K unique tokens) as a general medical embedding, and build 3 specific embeddings: embed-mimic-nursing (223 K notes, 37 K unique tokens), embed-mimic-radiology (522 K notes, 24 K unique tokens), and embed-mimic-discharge (59 K notes, 31 K unique tokens).

## Results

We go through our four scenarios, presenting results and discussing their implications.

### A fully customized system

We train custom models on the three datasets that contain sufficient PHI for a fully trained model. The fully customized results are given in Table 2, indicating that state-of-the-art systems provide protected health information recall >97%.

**Table 2:** Clinical note de-identification using fully customized systems, showing >97% recall of protected health information

Dataset	Recall (%)	Precision (%)	F1
i2b2-2014	99.1	85.7	91.7
i2b2-2006	99.6	90.7	94.9
mimic-discharge	97.1	96.3	96.7

To illustrate the challenges remaining in even the best-performing de-identification scenario, we consider the errors on the i2b2-2014 model. From the 15,201 PHI elements in the evaluation set, the model classified 15 K true positives, 116 false negatives, and ~ 2.5 K false positives.

We focus on the errors in Name, as the most identifying field. Name had 14 false negatives, i.e. undiscovered PHI: 3 doctor initials, 1 patient single-letter suffix (“I” as in “John Smith I”), 1 dataset-mislabeled apostrophe-s, and 9 names. All 9 names were dictionary words (“...saw Guy in PT”, “Strong to cover for...”), showing remaining challenges in automated de-identification.

False positives remove information useful to researchers; they are worth reviewing both to see what is being unnecessarily lost, and to get an intuition for the workings of the algorithm. The Name false positives included medical terms similar to names (“thrombosed St. Jude valve”, “chronic indwelling Foley”, “per Bruce Protocol”, “Zoran”), which could be corrected using heuristics based on medical terminology; errors due to overreliance on sentence structure, e.g. the second word after a title being labeled a name (“awoke” was labeled in “Ms Mitchel awoke feeling...”); and misspellings creating non-dictionary words (“Interesetd (sic) in quitting smoking”).

### An off-the-shelf system

Our next deployment scenario is an organization using an off-the-shelf system with no customization. We use our custom models from the previous section. We test each model on the datasets with compatible labeling schemes, reporting recall/precision/F1 for all PHI types combined. We then present a full cross-dataset analysis using Name only.

The i2b2-2014 model tested on physionet yields 76.6/60.5/67.6. Error analysis shows that 272 of the 441 false negatives (i.e. missed PHI) are of type Location, and consist mainly of “MICU”, “PMICU”, “cath lab”, and similar. Investigation revealed that these initials appear only in physionet, not i2b2-2014, thus providing a good example of an off-the-shelf system missing local jargon. Dropping Location from the analysis yields an improved 89.1/59.8/71.6; this improvement shows that a real deployment could consider using an off-the-shelf model together with heuristics (such as a list of local PHI abbreviations) gleaned from a manual error analysis.

The mimic-discharge model tested on mimic-radiology yields 65.7/90.9/76.2. Error analysis shows that 595 of the 597 false negatives are of type Id; of these errors, 577 are the 7-digit record number at the top of every note. This error is again dataset-specific and easily fixed with a simple heuristic. Including the heuristic yields 99.4/95.2/97.3, on par with a custom model.

The mimic-discharge model test on mimic-echo yields 99.7/98.7/99.2, on par with a custom model and thus showing that de-identification of some datasets can be accomplished without a customized system.

Table 3 presents results for a full cross-dataset analysis using Name only. Results show more variability than in the fully customized scenario, although recall is always above 90%. The exception is i2b2-2006’s experiments; error analysis showed that the made-up names used in the dataset [26] (“FREIERMFETHBREUN, HILDERINEMI”, “DALEJESC, OIE”) contained little information, hampering the model’s ability to learn.

**Table 3:** Off-the-shelf systems recall >90% of Names, with the exception of experiments using the i2b2-2006 dataset

		Train on		
Test on		i2b2-2014	mimic-discharge	i2b2-2006
	i2b2-2014	98.8/94.6/96.7	95.7/85.6/90.3	86.2/85.2/85.7
	physionet	92.9/73.1/81.8	94.3/70.6/80.7	69.0/78.6/73.4
	mimic-radiology	92.9/85.7/89.1	97.0/87.0/91.7	78.2/75.8/76.8
	mimic-discharge	92.5/85.4/88.8	97.9/85.2/91.0	79.1/85.4/82.1
	mimic-echo	95.5/61.4/74.7	99.6/86.6/92.6	54.2/20.3/29.0
	i2b2-2006	87.5/86.7/87.0	76.9/85.1/80.8	97.0/97.2/97.1

### Partial customization using a small number of labeled examples

For the large labeled dataset “A” we use i2b2-2014; for the partially labeled dataset “B” we run experiments using physionet, mimic-radiology, and mimic-discharge.

**Fig. 2:**
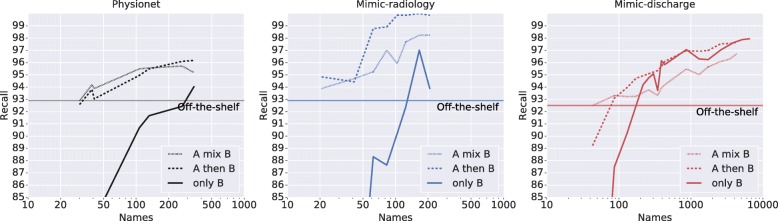
System performance as a function of number of labeled names. In each subfigure, an off-the-shelf system trained on dataset “A” (i2b2-2014) is partially customized using labeled examples from the target dataset “B”, then the system is evaluated on “B”. Training a system from scratch on “only B” is provided for comparison

Figure 2 shows recall as a function of the number of Names in “B”. “A mix B” roughly follows “A then B”. From the “only B” curves for physionet and mimic-radiology, it is clear that we run out of data long before the models have finished learning; the datasets benefit greatly when supplemented from “A then B”. For mimic-radiology, using ~ 20 labeled examples in “A then B” has raised the performance over the off-the-shelf result from the previous section. Although this small number seems surprising, radiology notes have a relatively uniform structure easily learned from the context surrounding the examples. For the more varied physionet, ~ 100 labeled examples are necessary to achieve the same gain.

The larger mimic-discharge shows that over ~ 500 labels, one can train solely on “B”. Around 1000 labels, performance reaches that of fully customized (97.1%); around this number, one sees diminishing returns from further labeling effort. At ~ 80 labels, the “A then B” model is already better than the off-the-shelf system, again demonstrating the usefulness of even small labeled datasets.

### Partial customization using a large number of unlabeled examples

We train using i2b2-2014 as dataset “A” with different token embeddings, and select test dataset “B” from physionet, mimic-radiology, mimic-discharge, and mimic-echo. For each choice of “B”, we evaluate three different token embeddings: the generic GloVe embedding, embed-mimic, and the embed-mimic-* that matches “B”. (For physionet, which contains nursing data, we use a subset of nursing data from the much larger mimic corpus). For the mimic datasets, we give recall/precision/F1 on Name PHI; for physionet, whose labeling matches i2b2-2014’s, we also report on all types.

Table 4 shows that switching from GloVe to embed-mimic improves results for all datasets. Using a matching embedding resulted in equivalent or decreased performance. Studying the false negatives in the specific embeddings reveals that most (70–100%) were from out-of-vocabulary tokens, showing that these specific embeddings did not encompass a large enough vocabulary. Thus, organizations can gain significant improvement from this partial customization technique, but only if they are able to provide a sufficiently large corpus.

**Table 4:** Performance of an i2b2-2014 model with custom embedding tested on 4 different datasets

Test on	PHI type	Embedding		
		GloVe	embed-mimic	Matching embedding
physionet	All	76.2/61.3/67.9	81.8/64.1/71.8	76.9/62.4/68.9 - embed-mimic-nursing
physionet	Name	92.9/73.1/81.8	95.5/81.8/88.1	91.0/73.4/81.3 - embed-mimic-nursing
mimic-radiology	Name	92.9/85.7/89.1	97.2/85.9/91	92.0/87.2/89.4 - embed-mimic-radiology
mimic-discharge	Name	92.5/85.4/88.8	93.4/89.8/91.6	92.1/86.3/89.1 - embed-mimic-discharge
mimic-echo	Name	95.5/61.4/74.7	98.7/64.4/77.3	too small to build embedding

## Discussion

In our work we follow the same datasets through various levels of system customization, thereby creating a robust picture of the performance a health organization can expect from a de-identification system under different scenarios. This is while obtaining results on par with the available literature at comparable data points [5, 6, 20, 37].

Automated de-identification systems can be used to add an extra layer of security while working with trusted research collaborators, or to minimize exposure of PHI to human annotators who will complete the de-identification task. Automated de-identification can also play a role in a HIPAA-compliant data release, with the additional step of “Expert Determination,” wherein a human expert in the field determines if the de-identification process has ensured that “the risk is very small that the information could be used ... to identify an individual.” [25] Selecting a “sufficient” level of performance for these applications remains a question of balancing the resources required to de-identify to a certain privacy level, the analytic utility of the resulting dataset for researchers, and the risk of re-identifying an individual [40]. Future work should focus on taking lessons learned from real-world deployments and strive to establish metrics that incorporate these concerns.

## Conclusions

Based on our results, we present broad guidelines to inform an organization’s approach to de-identification using machine learning.

Organizations able to label on the order of 10 K PHI examples can expect their fully customized system to have a recall of 97–99%. Organizations also have the control to fine-tune the balance between recall and precision.

Organizations should try using an off-the-shelf system before committing to customization. Although performance varied widely, our experiments showed that recall can be dramatically improved (to 89–99%) with simple heuristics gleaned from manual error analysis.

Organizations with the resources to provide a small amount of labeled data will benefit from partial customization. Labeling even a small amount of PHI, ~ 20 to ~ 80 examples, will raise system performance over an off-the-shelf solution. Labeling ~ 1000 PHI will give results on par with full customization.

Organizations can avoid the cost and privacy concerns of labeling data, yet still gain in performance over off-the-shelf-systems, by creating a custom embedding using a large set of their unlabeled data.

These guidelines generalize from results on available datasets, and thus cannot provide performance guarantees. In practice one can ensure better baseline performance with additional de-identification techniques, such as adding organization-specific or generic heuristics, or enhancing a pure machine learning system with a human in the loop.

Our results highlight the need for additional medical corpora with identical labeling schemes. Contributions of notes from a variety of healthcare systems, large and small, encompassing different jargon and distributions of identifiers, would go a long way towards the goal of building a truly generic, off-the-shelf system requiring no customization. Such a collection could also form a universally recognized benchmark for evaluating commercial offerings.

## Data Availability

The datasets supporting the conclusions of this article are available in the i2b2 repository, 10.1197/jamia.M2444, and the MIMIC-III Clinical Database, 10.13026/C2XW26. The Physionet Gold Standard corpus and its labeling following the i2b2-2014 guidelines are available at 10.1161/01.CIR.101.23.e215 and https://www.kaggle.com/google-health/deid-annotations respectively. Code is available at https://github.com/google/NeuroNER-CSPMC.

## Abbreviations

BiRNN: Bidirectional recurrent neural networks
GloVe: Global Vectors for Word Representation
HIPAA: Health Insurance Portability and Accountability Act
i2b2: Informatics for Integrating Biology & the Bedside
MIMIC: Medical Information Mart for Intensive Care
NLP: Natural Language Processing
PHI: Protected Health Information
